# Melatonin Receptors Agonistic Activities of Phenols from *Gastrodia elata*

**DOI:** 10.1007/s13659-019-0213-2

**Published:** 2019-06-07

**Authors:** Si-Yue Chen, Chang-An Geng, Yun-Bao Ma, Ji-Jun Chen

**Affiliations:** 1 State Key Laboratory of Phytochemistry and Plant Resources in West China, Kunming Institute of Botany, Yunnan Key Laboratory of Natural Medicinal Chemistry, Chinese Academy of Sciences, Kunming, 650201 China; 2 University of Chinese Academy of Sciences, Beijing, 100049 China

**Keywords:** *Gastrodia elata*, Gastropolybenzylols, Melatonin receptors

## Abstract

**Abstract:**

*Gastrodia elata* is a famous traditional Chinese herb with medicinal and edible application. In this study, three new polybenzyls, gastropolybenzylols G-I (**1–3**) were isolated from the EtOAc extract of *G. elata.* Their structures were identified by extensive spectroscopic analyses involving HRESIMS, UV, IR, 1D and 2D NMR. Compound **1** showed agonistic effects on MT_1_ and MT_2_ receptors with agonistic rates of 55.91±4.84% and 165.13±5.65% at the concentration of 0.5 mM, respectively, and an EC_50_ value of 76.24 *μ*M on MT_2_ receptor.

**Graphic Abstract:**

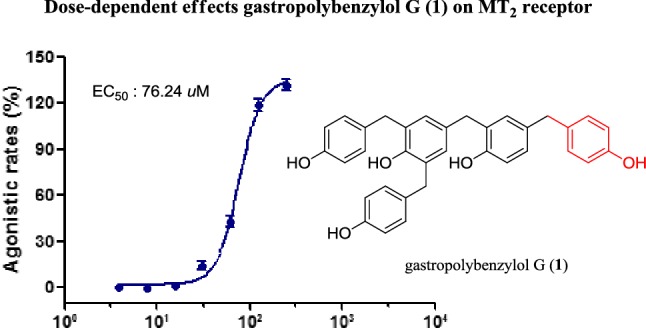

**Electronic supplementary material:**

The online version of this article (10.1007/s13659-019-0213-2) contains supplementary material, which is available to authorized users.

## Introduction

**Fig. 1:**
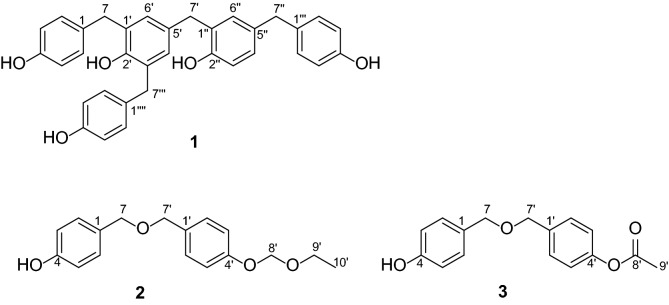
Structures of compounds **1**–**3**

*Gastrodia elata*, a saprophytic herb from orchid family, mainly distributed in East Asia, Southeast Asia and Oceania. The main constituents of *G. elata* are phenolics, polysaccharides, sterols and organic acids [1]. As a famous traditional Chinese medicine (TCM), the tuber of *G. elata* is commonly used for the treatment of diverse mental diseases, involving insomnia [2, 3], depression [4–7], epilepsy, convulsion [8, 9], and Alzheimer’s disease [10, 11]. Plenty of pharmacological investigation suggested that *G. elata* had anti-hypertension [12], anti-tumor [13], anti-virus [14, 15] and anti-inflammation [16, 17] effects. Most of pharmacological components are 4-hydroxybenzyl alcohol, 4-hydroxybenzaldehyde, vanillin, 1,3-bis(4-hydroxybenzyl) citrate, 1-(4-beta-d-glucopyranosyloxybenzyl) citrate and parishin B [18]. However few reports about the psychoactive effects of polybenzyls were published [19–22]. In this study, the EtOAc extract of *G. elata* was found to activate MT_2_ receptor with an agonistic rate of 105.38% at the concentration of 102.22 *μ*g/mL. In order to characterize the active constituents, three new compounds (**1–3**, Fig. 1) were obtained by various column chromatography. Herein, we report their isolation, structural elucidation and agonistic activities on melatonin (MT_1_ and MT_2_) receptors.

## Results and Discussion

### Structure Elucidation

**Fig. 2:**
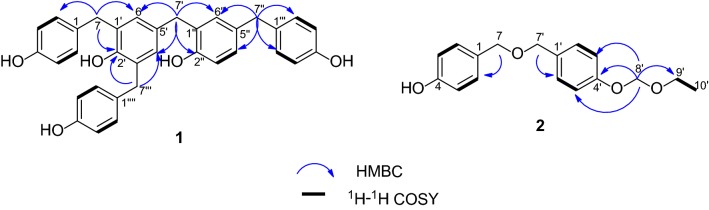
The key ^1^H-^1^H COSY and the HMBC correlations of compounds **1** and **2**

Compound **1** had a molecular formula of C_34_H_30_O_5_ with 20 degrees of unsaturation, which was deduced from HRESIMS at *m/*z 517.2040 [M−H]^−^ (calcd 517.2020). The UV absorptions at 281 and 254 nm, and the IR absorptions at 3426, 1631, 1613, 1511 and 1439 cm^−1^ suggested the presence of hydroxyl and phenyl groups. In the ^1^H-NMR spectrum, three sets of protons [*δ*_H_ 7.01 (4H, d, *J* = 8.3 Hz, H-2, H-6, H-2′‴, H-6′‴), 6.67 (4H, overlap, H-3, H-5, H-3′‴, H-5′‴); 6.93 (2H, d, *J* = 8.4 Hz, H-2‴, H-6‴), 6.67 (2H, overlap, H-3‴, H-5‴)], and combining with ^1^H-^1^H COSY correlations of H-2/H-3, H-6/H-5, H-2′‴/H-3′‴, H-6′‴/H-5′‴, and of H-2‴/H-3‴, H-6‴/H-5‴ indicated the existence of 1,4-substituted aromatic rings. One set of protons [*δ*_H_ 6.85 (1H, brs, H-6″), 6.84 (1H, *J* = 8.4, 2.0 Hz, H-4″), 6.67 (1H, overlap, H-3″)], and combining with ^1^H-^1^H COSY correlation of H-3″/H-4″ manifested the presence of a 1,2,4-substitued benzene ring. Besides, two *meta*-coupled protons [*δ*_H_ 6.77 (1H, d, *J* = 2.0 Hz, H-4′) and 6.76 (1H, d, *J* = 2.0 Hz, H-6′)], four methylenes [*δ*_H_ 3.79 (4H, s, H-7, H-7‴), 3.75 (2H, s, H-7′), 3.68 (2H, s, H-7″)] were well recognized (Table 1). Compared with 4-[[2-hydroxy-5-(methoxymethyl) phenyl] methyl]-2,6-bis [(4-hydroxyphenyl) methyl] phenol [19], compound **1** had an extra *para*- hydroxybenzyl [*δ*_H_ 6.93 (2H, d, *J* = 8.4 Hz, H-2‴, H-6‴), 6.67 (2H, overlap, H-3‴, H-5‴), 3.68 (2H, s, H-7″); *δ*_C_ 156.4 (C-4‴, s), 134.5 (C-1‴, s), 130.8 (C-2‴, C-6‴, d), 116.1 (C-3‴, C-5‴, d), 41.3 (C-7″, t)], but with the absence of a methoxymethyl group. Based on the HMBC correlations from H-7″ (*δ*_H_ 3.68, s) to C-2‴, C-6‴ (*δ*_C_ 132.1, d), C-4″ (128.5, d), C-6″ (*δ*_C_ 132.2, d), the linkages of C-7″ to C-5″ and C-1‴ were established (Fig. 2). Thus, compound **1** was elucidated and named as gastropolybenzylol G.

**Table 1:** ^1^H-NMR (400 MHz) and ^13^C-NMR (100 MHz) data of compound **1** in CD_3_OD

No.	*δ*_H_ (*J* in Hz)	*δ*_C_	No.	*δ*_H_ (*J* in Hz)	*δ*_C_
1	–	133.6 (s)	3″	6.67 (1H, overlap)	116.0 (d)
2	7.01 (1H, d, 8.3)	130.9 (d)	4″	6.84 (1H, 8.4, 2.0)	128.5 (d)
3	6.67 (1H, overlap)	116.0 (d)	5″	–	133.8 (s)
4	–	156.3 (s)	6″	6.85 (1H, brs)	132.2 (d)
5	6.67 (1H, overlap)	116.0 (d)	7″	3.68 (2H, s)	41.3 (t)
6	7.01 (1H, 8.3)	130.9 (d)	1‴	–	134.5 (s)
7	3.79 (2H, s)	36.0 (t)	2‴	6.93 (1H, d, 8.4)	130.8 (d)
1′	–	129.6 (s)	3‴	6.67 (1H, overlap)	116.1 (d)
2′	–	154.2 (s)	4‴	–	156.4 (s)
3′	–	129.5 (s)	5‴	6.67 (1H, overlap)	116.1 (d)
4′	6.77 (1H, d, 2.0)	132.0 (d)	6‴	6.93 (1H, d, 8.4)	130.8 (d)
5′	–	134.4 (s)	7‴	3.79 (2H, s)	35.9 (t)
6′	6.76 (1H, d, 2.0)	132.0(d)	1′‴	–	133.9 (s)
7′	3.75 (2H, s)	35.9 (t)	2′‴	7.01 (1H, d, 8.3)	130.9 (s)
1″	–	129.4 (s)	3′‴	6.67 (1H, overlap)	116.0 (d)
2″	–	154.1 (s)	4′‴	–	154.2 (s)
			5′‴	6.67 (1H, overlap)	116.0 (d)
			6′‴	7.01 (1H, d, 8.3)	130.9 (d)

Compound **2** was assigned a molecular formula of C_17_H_20_O_4_ with 8 degrees of unsaturation according to the HRESIMS at *m/z* 333.1344 [M+HCOO]^−^ (calcd for 333.1344). The UV (274, 247 and 226 nm) and the IR (3423, 1613 and 1513 cm^−1^) absorptions showed the presence of hydroxyl and phenyl groups. Compared with 4,4′-hydroxybenzyl ether [23], compound **2** had an additional ethoxymethoxyl group [*δ*_H_ 5.21 (2H, s, H-8′), 3.70 (2H, q, H-9′), 1.18 (3H, t, H-10′); *δ*_C_ 94.2 (C-8′, t), 65.2 (C-9′, t), 15.5 (C-10′, q)] in accordance with the HMBC correlation from H-8′ to C-9′. The HMBC correlation from H-8′ (*δ*_H_ 5.21, 2H, s) to C-4′ (*δ*_C_ 158.5, s) indicated the ethoxymethoxyl group at C-4′. Therefore, compound **2** was identified and named as gastropolybenzylol H.

Compound **3** showed a molecular formula of C_16_H_16_O_4_ with 9 degrees of unsaturation according to the [M+H]^+^ ion at *m/z* 273.1122 (calcd for 273.1121). The UV absorptions at 275, 250 and 229 nm suggested the existence of phenolic structure and the IR absorptions at 3430, 1703, 1614 and 1518 cm^−1^ manifested the presence of hydroxyl, carbonyl and phenyl groups. Compared with 4,4′-hydroxybenzyl ether [23], compound **3** had an extra acetyl group [*δ*_C_ 172.9 (s, C-8′), 21.0 (q, C-9′)]. Thus, compound **3** was determined as the acetylated derivative of 4,4′-hydroxybenzyl ether and named as gastropolybenzylol I.

The known compounds were identified as 4, 4′-dimethoxy-dibenzyl ether [24] (**4**), 4,4′-diethoxybenzyl ether [25] (**5**), 4-[(4-(methoxymethyl) phenoxy] methyl] phenol [23] (**6**) and 4-[[4-[[4-(ethoxymethyl) phenoxy] methyl] phenoxy] methyl] phenol [26] (**7**).

### MT_1_ and MT_2_ Receptors Agonistic Activities

**Fig. 3:**
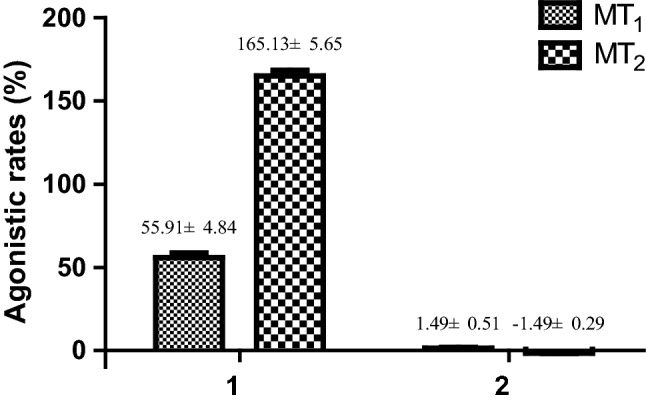
MT_1_ and MT_2_ receptors agonistic activities of compounds **1** and **2** (0.5 mM). Data are expressed as mean ± S.D., n=3. Melatonin was used as the positive control with EC_50_ values of 1.0 nM (MT_1_) and 25.0 nM (MT_2_)

**Fig. 4:**
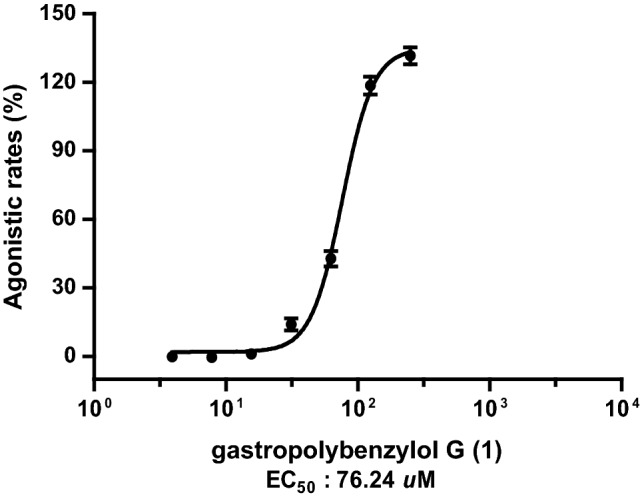
Dose-dependent effects gastropolybenzylol G (**1**) on MT_2_ receptor

Compounds **1** and **2** were evaluated for their agonistic activities on MT_1_ and MT_2_ receptors in *vitro*. Melatonin was used as the positive control. As showed in Fig. 3, compound **1** could activate MT_1_ and MT_2_ receptors with agonistic rates of 55.91±4.84 and 165.13±5.65% at the concentration of 0.5 mM. Due to **1** with better agonistic activities on MT_2_ receptor, further study provided an EC_50_ value of 76.24 *μ*M on MT_2_ receptor (Fig. 4).

## Experimental Section

### General Procedures

NMR spectra were undertaken by Avance III-400/III-600 spectrometers (Bruker, Bremerhaven, Germany). UV spectra were recorded on a UV-2401 equipment (Shimadzu, Kyoto, Japan). IR spectra were obtained on Bio-Rad FTS-135 (Hercules, California, USA) spectrometers using KBr pellets. LCMS-IT-TOF (Shimadzu, Kyoto, Japan) was used for detecting MS spectra. Thin-layer chromatography (TLC) analyses were performed on silica gel GF254 plates (Jiangyou, Chemical Co. Ltd., Yantai, China). Compounds were purified by silica gel (200–300 mesh, Qingdao Makall group Co. Ltd., Qingdao, China), Sephadex LH-20 (Amersham Biosciences, Sweden) column chromatography and HPLC using Shimadzu LC-CBM-20 system (Shimadzu, Kyoto, Japan) with an Agilent XDB-C_18_ column (9.4 × 250 mm, Agilient, California, USA). 10% H_2_SO_4_ in EtOH was applied for detecting spots by heating after sprayed. Melatonin as positive control was obtained from Damas-beta Co. Ltd. (Shanghai, China).

### Plant Material

*Gastrodia elata* Bl. was bought from Zhaotong in Yunnan Province of China, in September 2014, and was identified by Prof. Li-Gong Lei, Kunming Institute of Botany, CAS. A voucher specimen (No. 20141107) was deposited at the Laboratory of Anti-virus and Natural Medicinal Chemistry, Kunming Institute of Botany, CAS.

### Extraction and Isolation

Fresh rhizomes of *G. elata* (45.0 kg) were cut into slices and extracted with 90% aqueous ethanol (45 L ×3) at room temperature. The combined extract was concentrated under reduced pressure and partitioned between H_2_O and EtOAc. The EtOAc extract (88 g) was separated by silica gel column chromatography (CC) using gradient elution with EtOAc-CHCl_3_ (0:100, 5:95, 10:90, 20:80, 40:60, 100:0, *v/v*) as the mobile phase to yield five fractions (Fr. 1–Fr. 5). Fr. 2 (22 g) was separated by silica gel CC with EtOAc-petroleum ether (5:95, 10:90, 20:80, 40:60, 100:0) to yield five fractions (Fr. 2.1–Fr. 2.5). Then, Fr. 2.2 (3.4 g) was further separated by silica gel CC (Me_2_CO-petroleum ether, 3:97, 5:95, 10:90, 20:80, 50:50) to give five fractions (Fr. 2.2.1–Fr. 2.2.5). Compounds **2** (10 mg) and **5** (30 mg) were purified from Fr. 2.2.1 by HPLC on an Agilent XDB-C_18_ column with the elution of MeCN–H_2_O (85:15, t_*R*_ = 30 min) and MeCN-H_2_O (90:10, t_*R*_ = 27 min), respectively. Compound **4** (24 mg) was isolated from Fr. 2.3 by Sephadex LH-20 (MeOH-CHCl_3_, 50:50) and finally purified by HPLC with the mobile phase of MeCN-H_2_O (60:40, t_*R*_ = 23 min). Fr. 2.4 was purified from by Sephadex LH-20 (MeOH-CHCl_3_, 50:50) and HPLC on an Agilent XDB-C_18_ column with the elution of MeCN-H_2_O (60:40, t_*R*_ = 20 min) to yield compound **3** (3.5 mg). Fr. 3 was subjected to silica gel CC (Me_2_CO-petroleum ether, 5:95, 10:90, 20:80, 40:60, 100:0) to yield five fractions (Fr. 3.1–Fr. 3.5). Fr 3.1 was isolated by silica gel CC (Me_2_CO-CHCl_3_, 5:95, 10:90, 20:80, 40:60, 100:0), Sephadex LH-20 CC (MeOH-CHCl_3_, 50:50), from which compounds **6** (2 mg, MeCN-H_2_O, 60:40, t_*R*_ = 25 min) and **7** (37 mg, MeCN-H_2_O, 45:55, t_*R*_ = 20 min) were obtained by HPLC purification. Further purification on Fr. 3.5 yielded compound **1** (10 mg) by silica gel CC, Sephadex LH-20 CC (MeOH-CHCl_3_ 50:50) and semi-preparative HPLC (MeCN-H_2_O, 30:70, t_*R*_ = 40 min).

*Gastropolybenzylol G (****1****)*, pale yellow powder, UV (MeOH) *λ*_max_ (log *ε*): 281 (4.03), 254 (3.25) nm; IR (KBr) *ν*_max_: 3426, 1631, 1613, 1511, 1439, 1247, 1229 cm^−1^; HRESIMS *m*/*z* 517.2040 [M−H]^−^ (calcd 517.2020). ^1^H-NMR and ^13^C-NMR data showed in Table 1.

*Gastropolybenzylol H (****2****)*, yellow colloidal solid, UV (MeOH) *λ*_max_ (log *ε*): 275 (3.38), 248 (2.61), 226 (4.30) nm; IR (KBr) *ν*_max_: 3423, 1613, 1513, 1223, 1072, 1003 cm^−1^; HRESIMS *m*/*z* 333.1344 [M+COOH]^−^ (calcd 333.1344). ^1^H-NMR and ^13^C-NMR data showed in Table 2.

**Table 2:** ^1^H-NMR and ^13^C-NMR data of compounds **2** and **3** in CD_3_OD

No.	**2**		**3**	
	*δ*_H_ (500 MHz, *J* in Hz)	*δ*_C_ (125 MHz)	*δ*_H_ (600 MHz, *J* in Hz)	*δ*_C_ (150 MHz)
1	–	130.2 (s)	–	129.9 (s)
2	7.15 (1H, d, 8.6)	130.9 (d)	7.17(1H, d, 8.7)	131.2 (d)
3	6.75 (1H, d, 8.6)	116.1 (d)	6.85(1H, d, 8.7)	116.0 (d)
4	–	158.3 (s)	–	160.5 (s)
5	6.75 (1H, d, 8.6)	116.1 (d)	6.85(1H, d, 8.7)	116.0 (d)
6	7.15 (1H, d, 8.6)	130.9 (d)	7.17 (1H, d, 8.7)	131.2 (d)
7	4.40 (2H, s)	72.8 (t)	4.92 (2H, s)	71.2 (t)
1′	–	132.7 (s)	–	129.4 (s)
2′	7.24 (1H, d, 8.7)	130.6 (d)	7.15 (1H, d, 8.5)	130.6 (d)
3′	7.00 (1H, d, 8.7)	117.2 (d)	6.68 (1H, d, 8.5)	116.3 (d)
4′	–	158.5 (s)	–	158.6 (s)
5′	7.00 (1H, d, 8.7)	117.2 (d)	6.68 (1H, d, 8.5)	116.3 (d)
6′	7.24 (1H, d, 8.7)	130.6 (d)	7.15 (1H, d, 8.5)	130.6 (d)
7′	4.42 (2H, s)	72.4 (t)	4.85 (2H, s)	67.3 (t)
8′	5.21 (2H, s)	94.2 (t)	–	172.9 (s)
9′	3.70 (2H, q)	65.2 (t)	1.94 (3H, s)	21.0 (q)
10′	1.18 (3H, t)	15.5 (q)		

*Gastropolybenzylol I (****3****)*, white powder, UV (MeOH) *λ*_max_ (log *ε*): 275 (3.48), 250 (2.89), 229 (4.42) nm; IR (KBr) *ν*_max_: 3430, 1703, 1614, 1518, 1268, 1245, 1029, 830 cm^−1^; HRESIMS *m*/*z* 273.1117 [M+H]^+^ (calcd 273.1121). ^1^H-NMR and ^13^C-NMR data showed in Table 2.

### Bioassay of Agonistic Activities on MT_1_ and MT_2_ Receptors

Agonistic activities of compounds **1** and **2** were evaluated on HEK293 cell lines stably expressing the human melatonin MT_1_ and MT_2_ receptors. Referring to the previous study [27, 28], cells were cultivated by adding Dulbecco’s modified eagle medium, G418 (400 *μ*g/mL) and fetal bovine serum (10%) in 5% CO_2_ incubator at 37 °C. Then, cells were put in a Matrigel^®^ coated 96-well black plate at a density of 4 × 10^4^/well and proliferated in CO_2_ for 24 h. Wash Free Fluo-8 Calcium Assay Kit (HD Biosicences Co. Ltd., Hd03-0010) was used for detecting calcium flow assay. Flex Station 3 Benchtop Multi-Mode Micro Plate Reader was applied for recording and reading data at room temperature using specified settings (excitation wave length, 485 nm, emission wave length, 525 nm, emission cut-off, 515 nm). EC_50_ values were calculated by GraphPad Prism 5 software.

## Electronic supplementary material

Below is the link to the electronic supplementary material.

Supplementary material 1 (PDF 835 kb)
