# Supplementary material for: Melatonin Receptors Agonistic Activities of Phenols from Gastrodia elata
Source: Nat Prod Bioprospect. 2019 Jun 7;9(4):297–302. doi: 10.1007/s13659-019-0213-2 (PMC6646438; doi:10.1007/s13659-019-0213-2)

**Supplementary Materials**

# Melatonin receptors agonistic activities of phenols from *Gastrodia elata*

Si-Yue Chen[a,b], Chang-An Geng[a], Yun-Bao Ma[a] and Ji-Jun Chen[a,b *]

[a] State Key Laboratory of Phytochemistry and Plant Resources in West China, Kunming Institute of Botany, Chinese Academy of Sciences, Yunnan Key Laboratory of Natural Medicinal Chemistry, Kunming 650201, China

[b] University of Chinese Academy of Sciences, Beijing 100049, China

*Corresponding author. Kunming Institute of Botany, Chinese Academy of Sciences, Kunming 650201, P. R. China. Tel.: +86 871 65223265; Fax: +86 871 65227197. *E-mail address*: chenjj@mail.kib.ac.cn (Ji -Jun Chen).

**List of Supplementary material**

1D NMR, 2D NMR ,HRESIMS , UV and IR Spectra of compound **1** S1—S8
1D NMR, 2D NMR ,HRESIMS , UV and IR Spectra of compound **2** S9—S16
1D NMR, HRESIMS , UV and IR Spectra of compound **3** S17—S21

**S1**. $^{1}$H-NMR (150 MHz, $CD_3OD$) of compound **1**

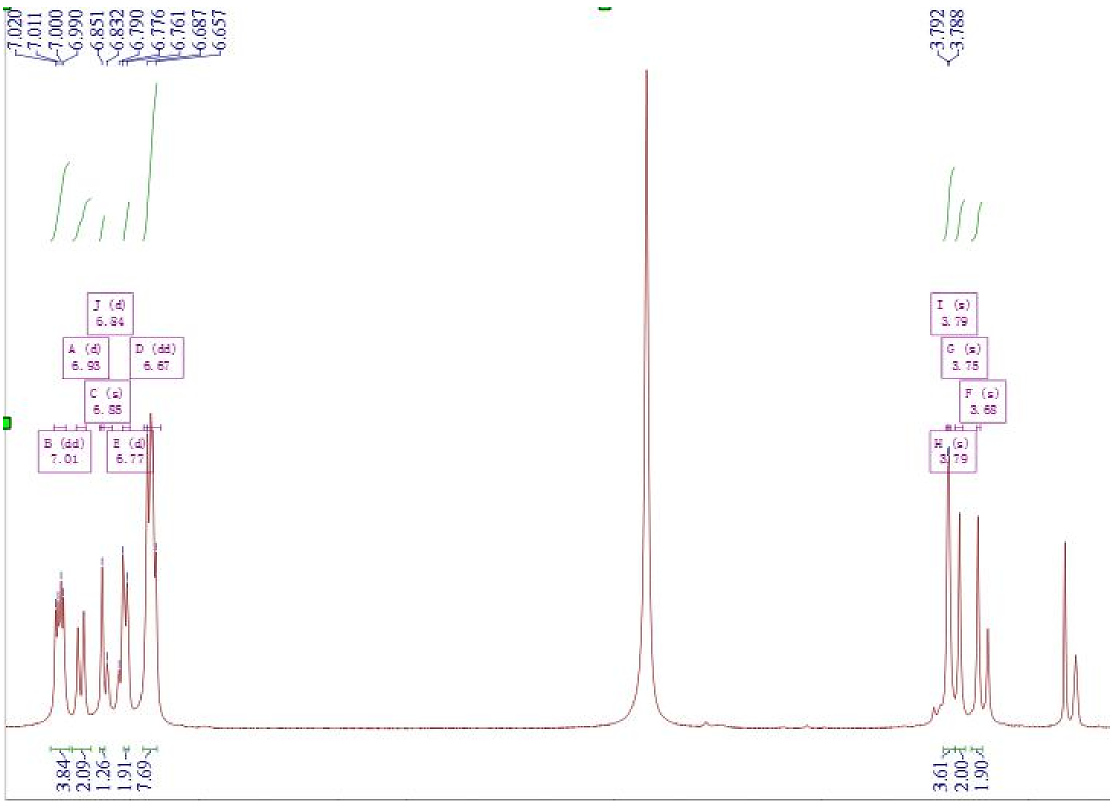


**S2**. $^{13}$C-NMR (150 MHz, $CD_3OD$) of compound **1**

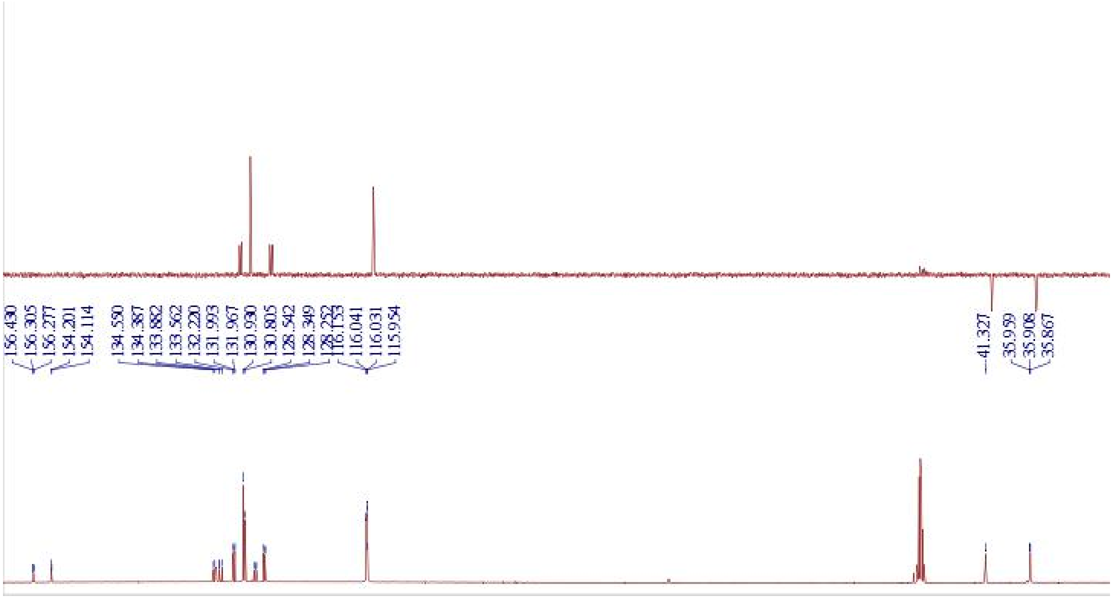

**S3**. HSQC (600 MHz, $CD_3OD$) of compound **1**

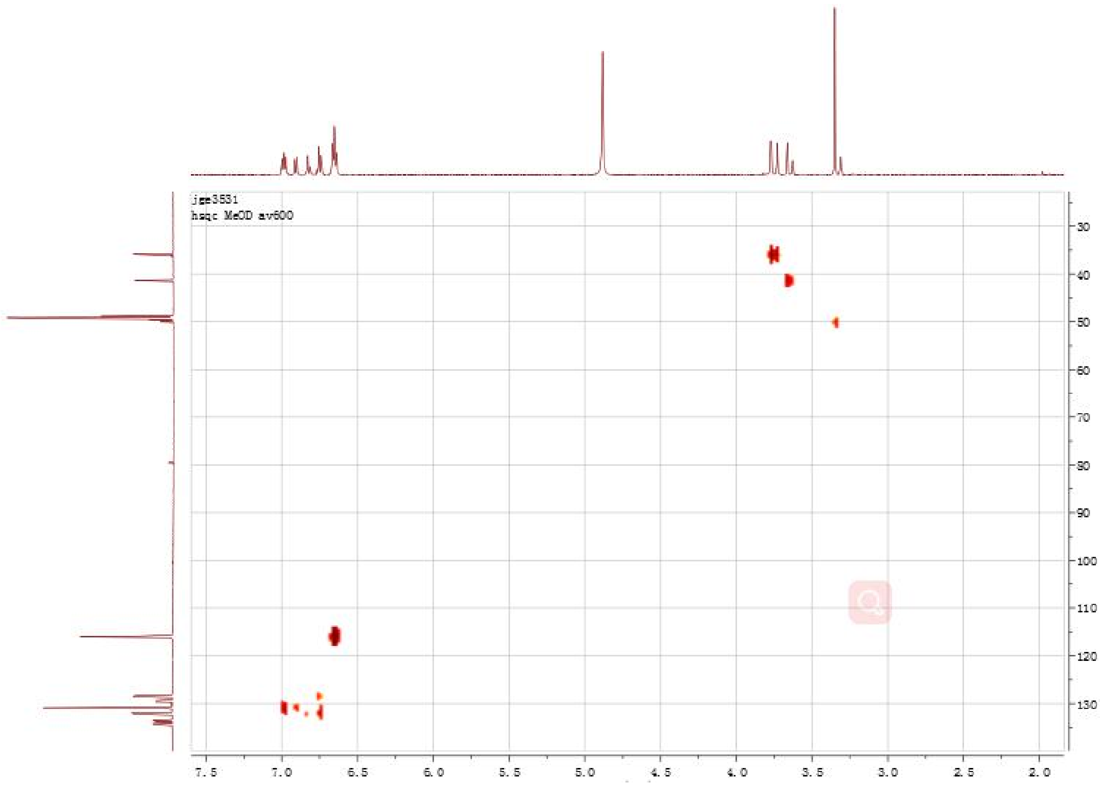


**S4**. $^1$H-$^1$H COSY (600 MHz, $CD_3OD$) of compound **1**

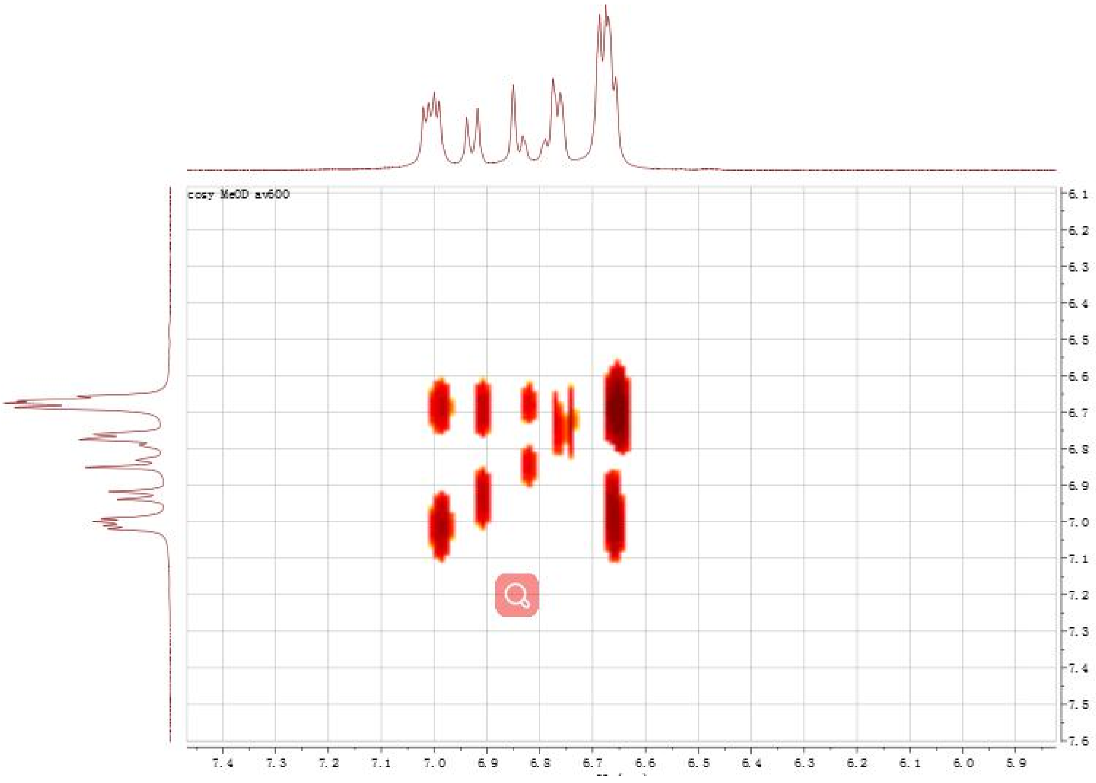

**S5**. HMBC (600 MHz, $CD_3OD$) of compound **1**

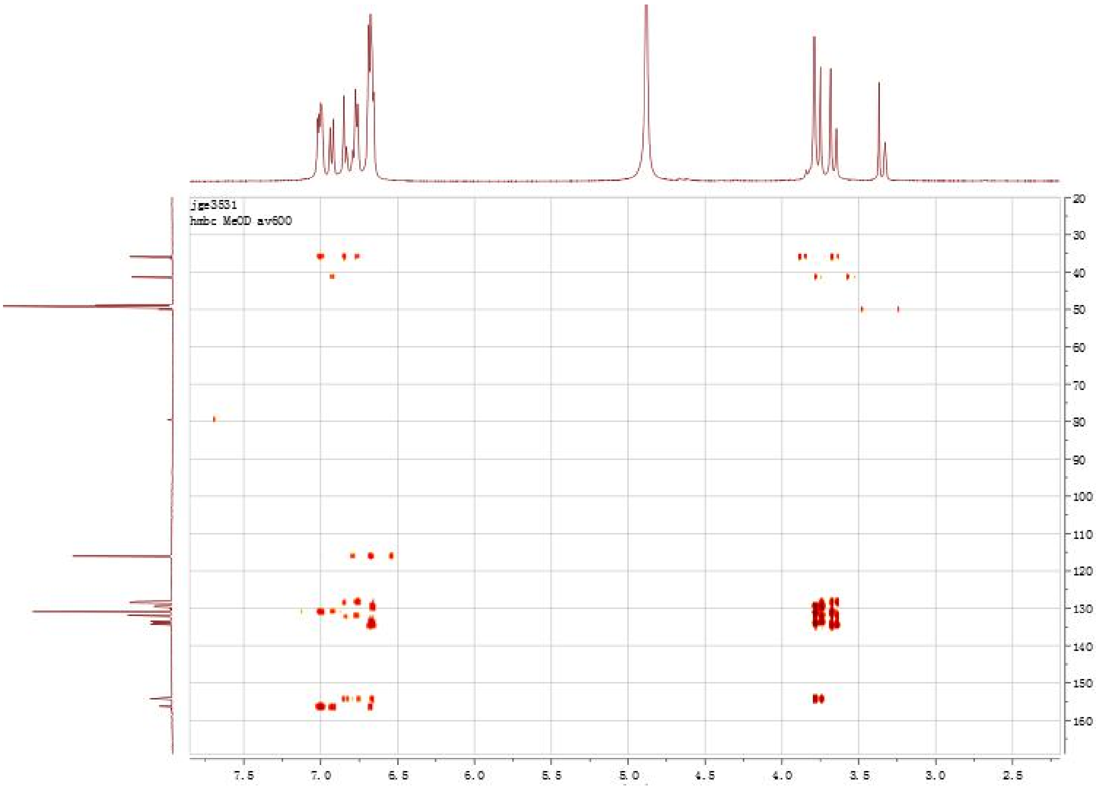

**S6.** HRESIMS spectra of compound **1**

| Elmt | Val. | Min | Max | Elmt | Val. | Min | Max | Use Adduct |
|---|---|---|---|---|---|---|---|---|
| H | 1 | 0 | 300 | S | 2 | 0 | 0 | H |
| C | 4 | 0 | 100 | | | | | HCOO |
| N | 3 | 0 | 0 | | | | | CH3COO |
| O | 2 | 0 | 40 | | | | | Cl |

| | | | | | |
|---|---|---|---|---|---|
| Error Margin (ppm): | 10 | DBE Range: | 0.0 - 20.0 | Electron Ions: | both |
| HC Ratio: | unlimited | Apply N Rule: | yes | Use MSn Info: | no |
| Max Isotopes: | all | Isotope RI (%): | 1.00 | Isotope Res: | 10000 |
| MSn Iso RI (%): | 75.00 | MSn Logic Mode: | AND | Max Results: | 500 |

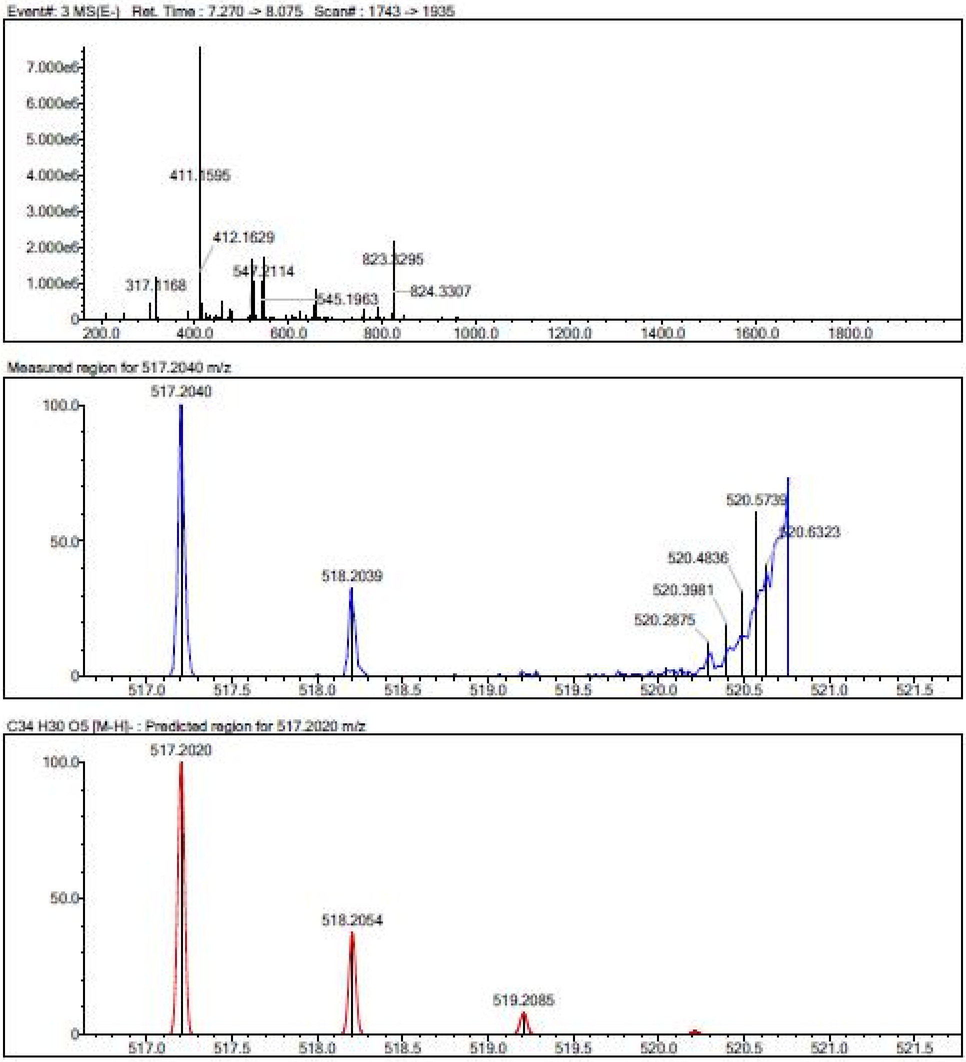


| Rank | Score | Formula (M) | Ion | Meas. m/z | Pred. m/z | Df. (mDa) | Df. (ppm) | Iso | DBE |
|---|---|---|---|---|---|---|---|---|---|
| 1 | 30.09 | C34 H30 O5 | [M-H]- | 517.2040 | 517.2020 | 2.0 | 3.87 | 32.42 | 20.0 |

**S7.** UV spectra of compound **1**

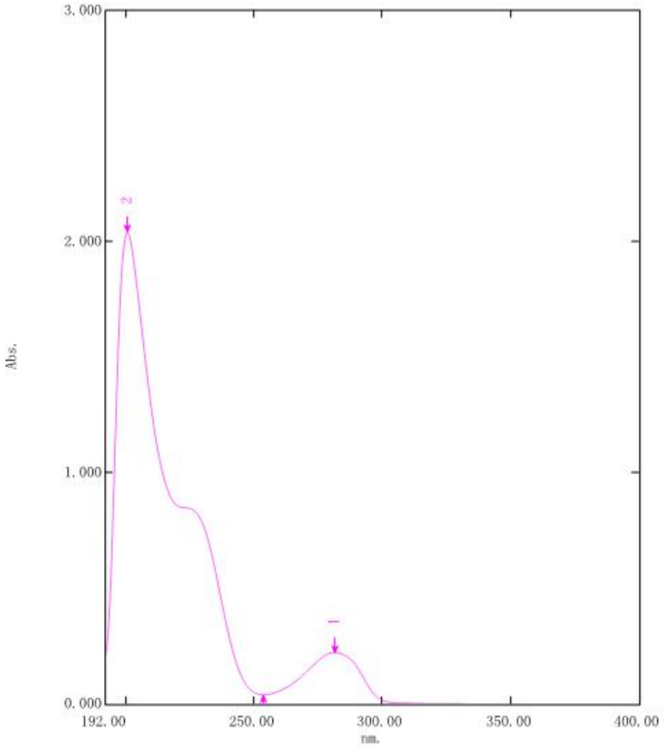


[测定属性]
波长范围(nm): 192.00 到 400.00
扫描速度: 中速
采样间隔: 0.2
自动采样间隔: 启用
扫描模式: 单个

[仪器属性]
仪器类型: UV-2700 系列
测定方式: 吸收值
狭缝宽: 5.0 nm
积分时间: 0.1 秒
光源转换波长: 323.0 nm
检测器单元: 直接
S/R 转换: 标准
阶梯校正: OFF

[附件属性]
附件: 无

[数据处理参数]
阈值: 0.0010000
点: 4
内插: 停用
平均: 停用

[样品准备属性]
重量: 0.33mg
体积: 1ml
稀释: 30倍
光程长: 10mm
附加信息: 2019/03/22 15:34:53
jge33531 CH3OH

| No. | P/V | 波长(nm) | Abs. | 描述 |
|---|---|---|---|---|
| 1 | ↑ | 281.40 | 0.221 | |
| 2 | ↑ | 200.60 | 2.039 | |
| 3 | ↓ | 253.80 | 0.038 | |

**S8.** UV spectra of compound **2**

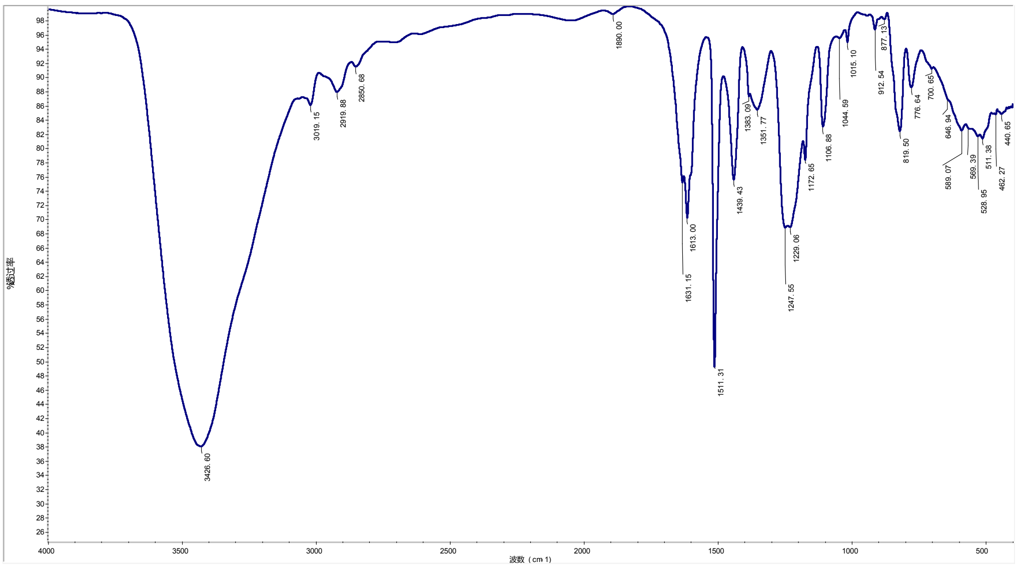


Sample Name: jge33531
KBr压片
采集时间: 星期二 3月 26 13:55:09 2019 (GMT+08:00)
仪器型号：NICOLET iS10
Software version: OMNIC 9.8.372

样品扫描次数: 16
背景扫描次数: 16
分辨率: 4.000
采样增益: 1.0
动镜速度: 0.4747
光阑: 80.00

**S9** $^{1}$H-NMR spectra of compound **2**

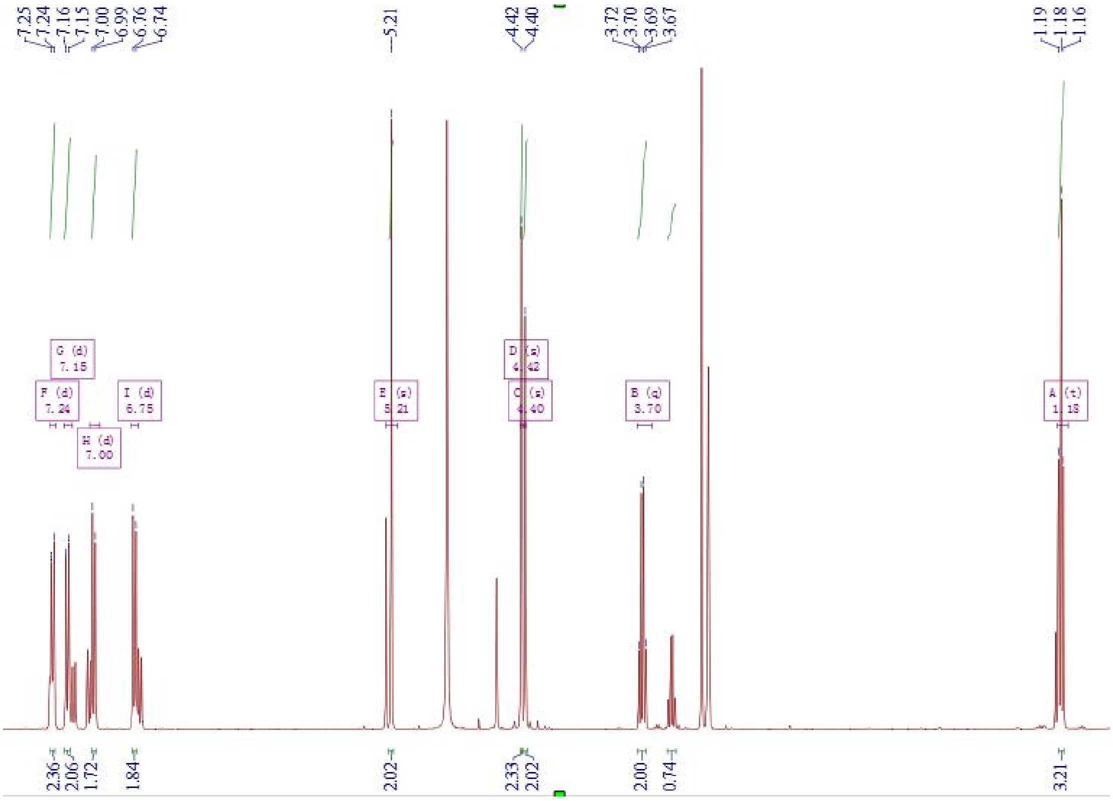


**S10.** $^{13}$C-NMR spectra of compound **2**

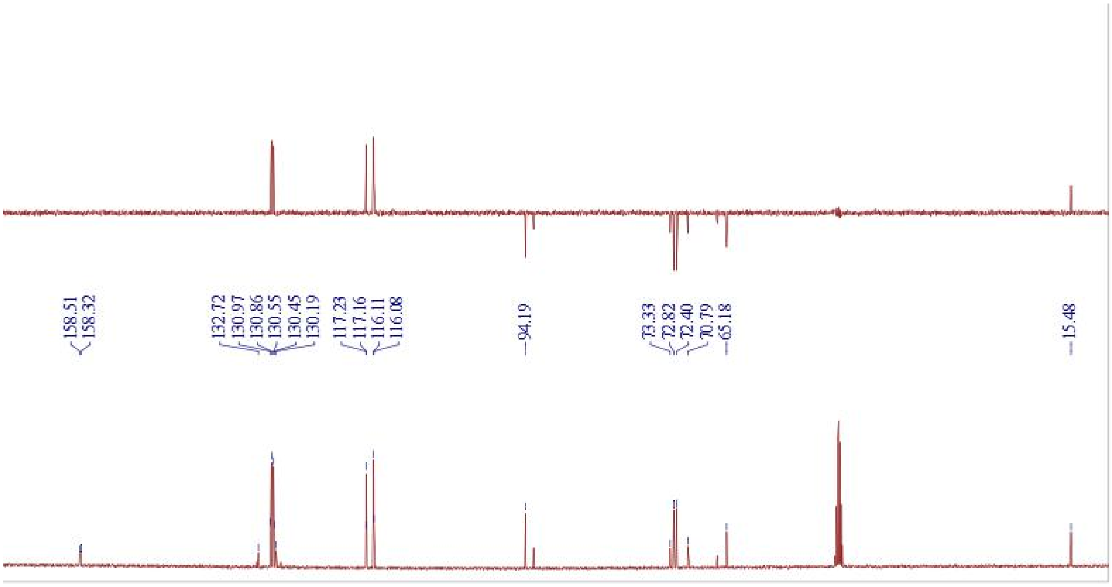

**S11.** SHQC spectra of compound **2**

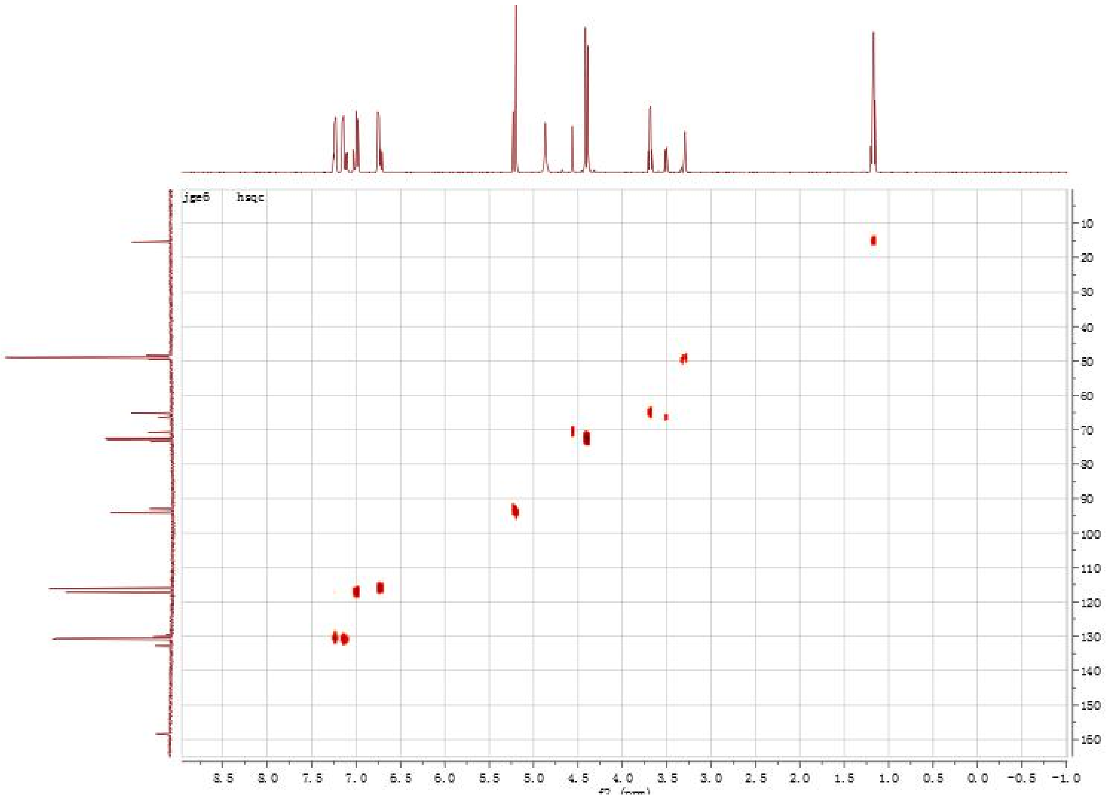


**S12.** $^{1}H$-$^{1}H$ COSY spectra of compound **2**

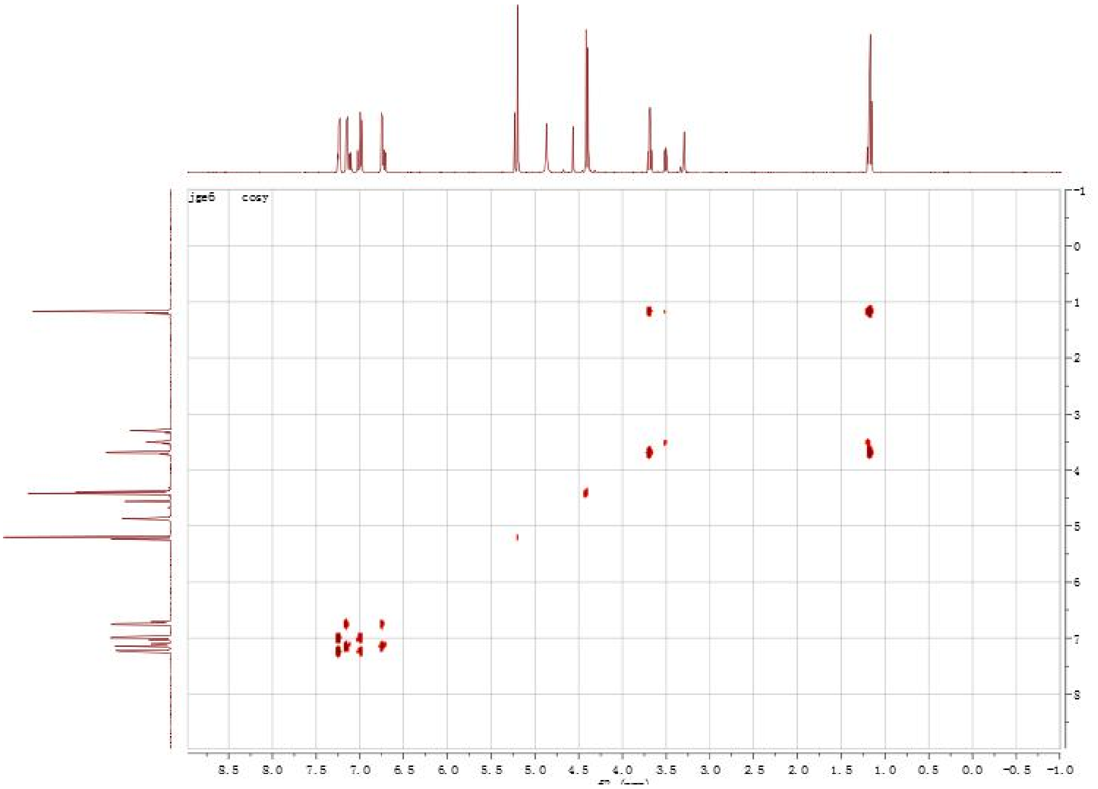

**S13.** HMBC spectra of compound **2**

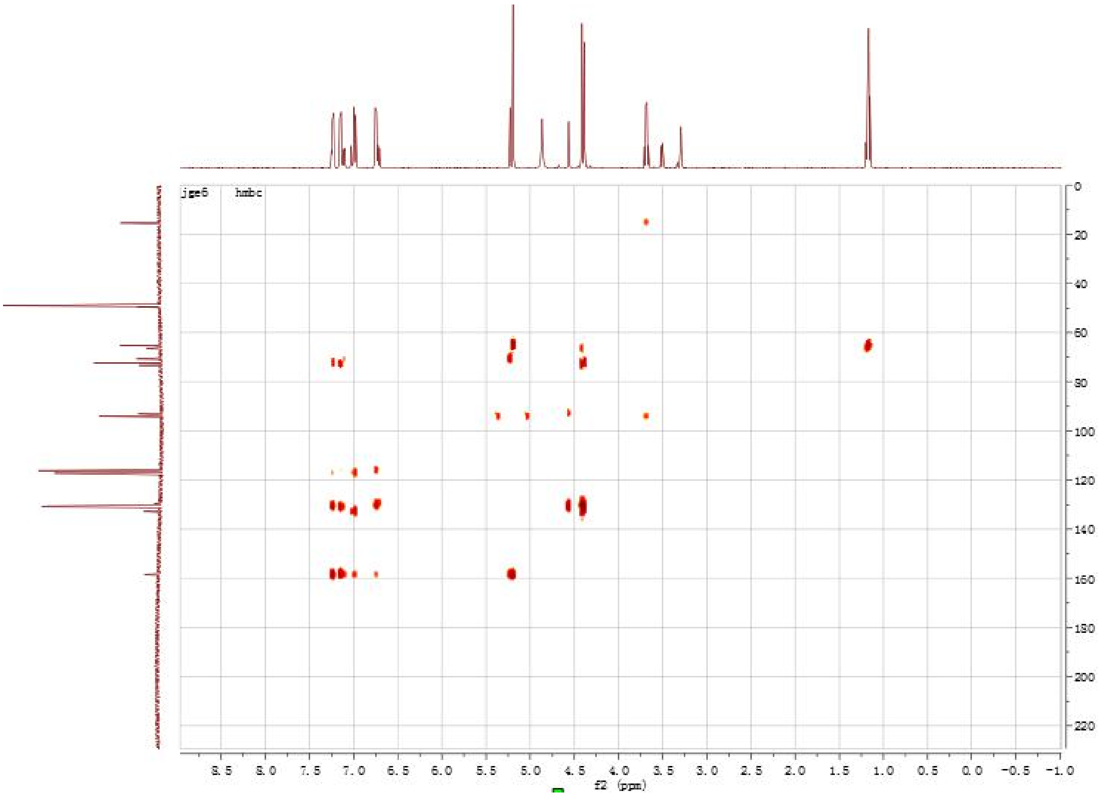

**S14.** HRESIMS spectra of compound **2**

| | | | | | |
|---|---|---|---|---|---|
| Error Margin (ppm): | 10 | DBE Range: | 0.0 - 20.0 | Electron Ions: | both |
| HC Ratio: | unlimited | Apply N Rule: | yes | Use MSn Info: | no |
| Max Isotopes: | all | Isotope RI (%): | 1.00 | Isotope Res: | 10000 |
| MSn Iso RI (%): | 75.00 | MSn Logic Mode: | AND | Max Results: | 500 |

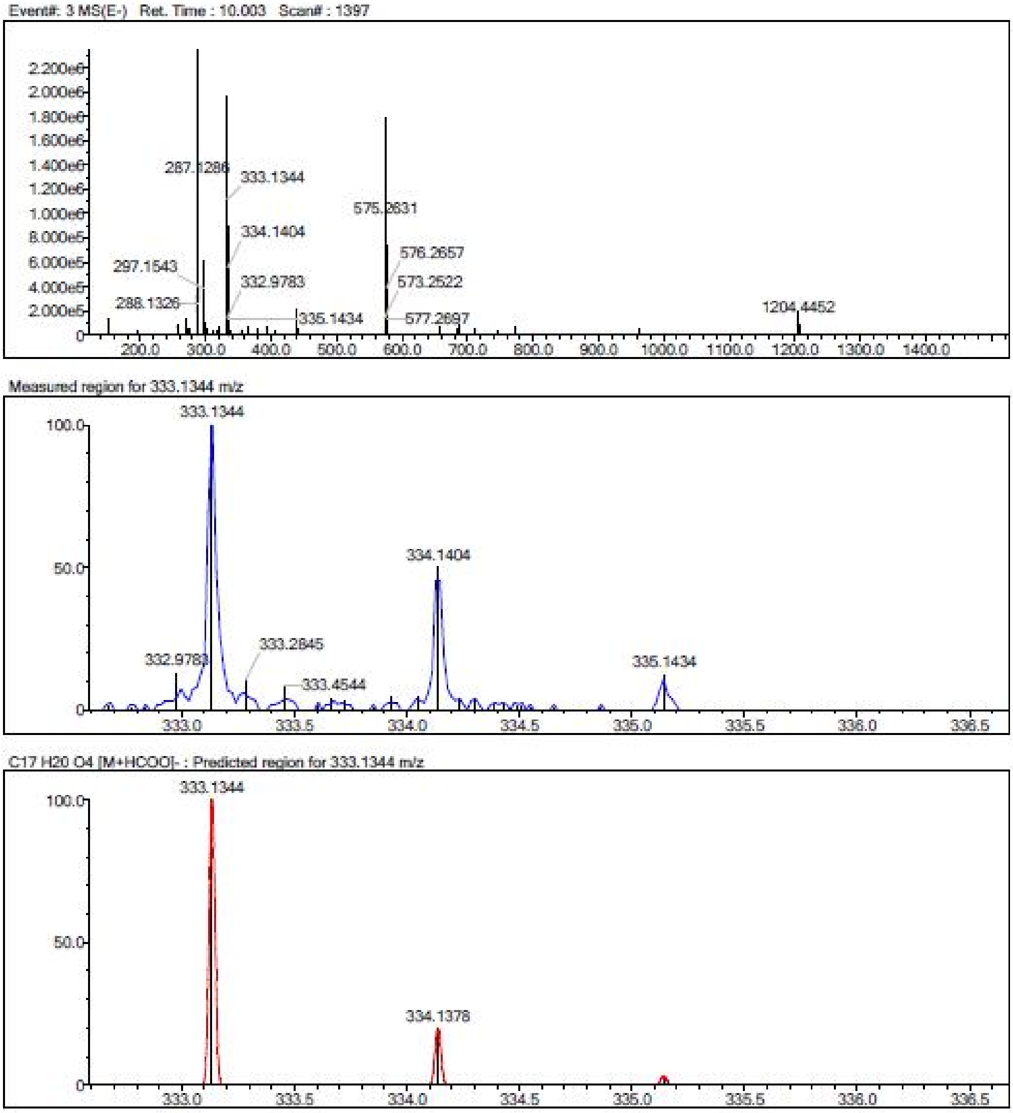


| Rank | Score | Formula (M) | Ion | Meas. m/z | Pred. m/z | Df. (mDa) | Df. (ppm) | Iso | DBE |
|---|---|---|---|---|---|---|---|---|---|
| 2 | 48.68 | C17 H20 O4 | [M+HCOO]- | 333.1344 | 333.1344 | 0.0 | 0.00 | 48.68 | 8.0 |

**S15.** UV spectra of compound **2**

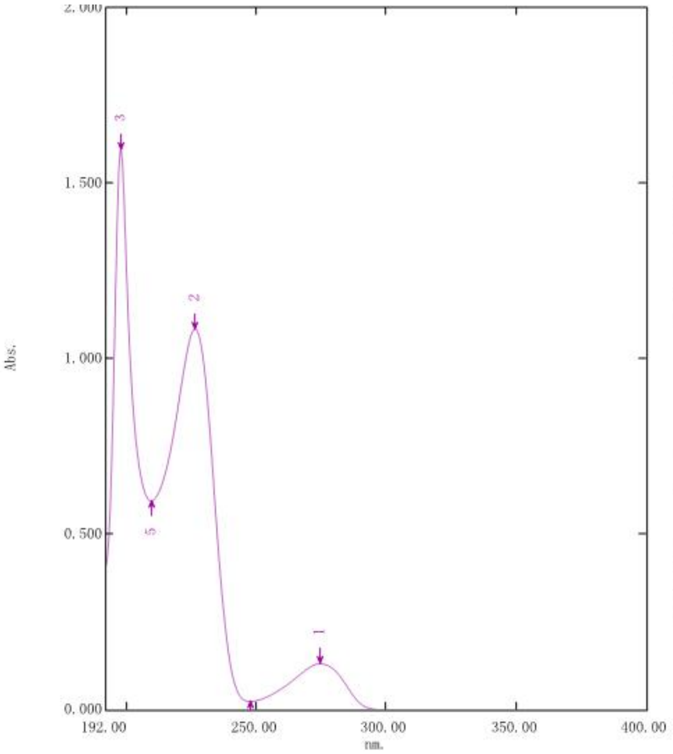


[测定属性]
波长范围(nm): 192.00 到 400.00
扫描速度: 中速
采样间隔: 0.2
自动采样间隔: 启用
扫描模式: 单个

[仪器属性]
仪器类型: UV-2700 系列
测定方式: 吸收值
狭缝宽: 5.0 nm
积分时间: 0.1 秒
光源转换波长: 323.0 nm
检测器单元: 直接
S/R 转换: 标准
阶梯校正: OFF

[附件属性]
附件: 无

[数据处理参数]
阈值: 0.0010000
点: 4
内插: 停用
平均: 停用

[样品准备属性]
重量: 0.47mg
体积: 1ml
稀释: 30倍
光程长: 10mm
附加信息: 2019/03/22 15:45:33
jge2 CH3OH

| No. | P/V | 波长(nm) | Abs. | 描述 |
|---|---|---|---|---|
| 1 | ↑ | 274.60 | 0.130 | |
| 2 | ↑ | 226.40 | 1.084 | |
| 3 | ↑ | 197.80 | 1.596 | |
| 4 | ↓ | 247.60 | 0.022 | |
| 5 | ↓ | 209.60 | 0.594 | |

**S16.** IR spectra of compound **2**

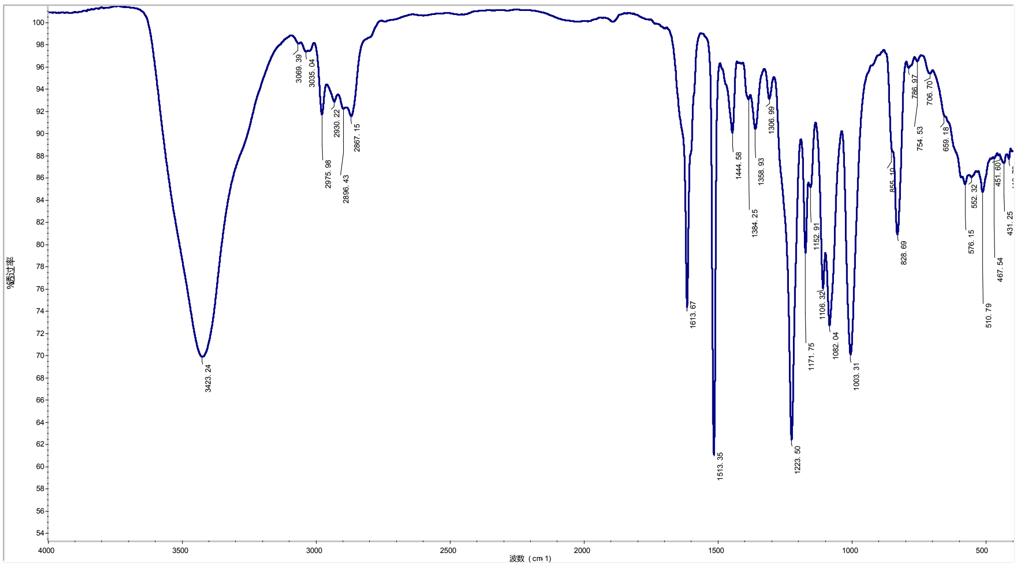


Sample Name: jge2
KBr压片
采集时间: 星期二 3月 26 11:01:29 2019 (GMT+08:00)
仪器型号：NICOLET iS10
Software version: OMNIC 9.8.372

样品扫描次数: 16
背景扫描次数: 16
分辨率: 4.000
采样增益: 1.0
动镜速度: 0.4747
光阑: 80.00

**S17.** $^{1}$H-NMR spectra of compound **3**

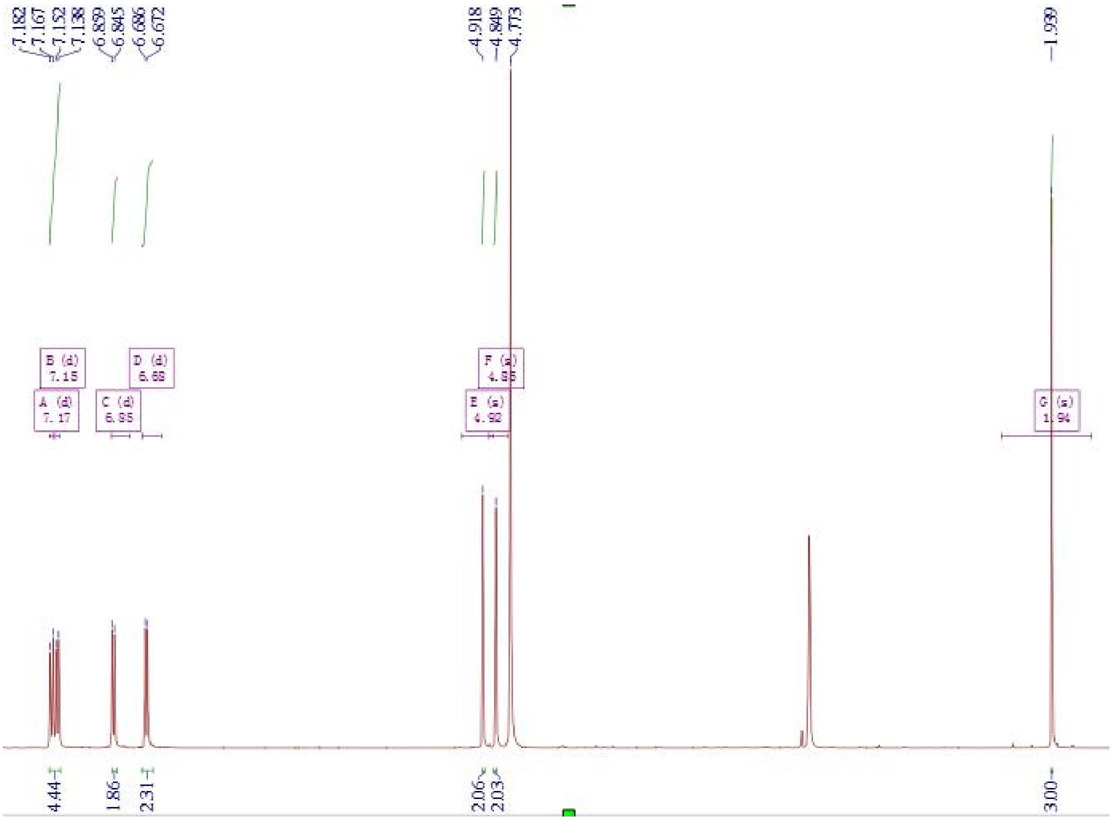


**S18.** $^{13}$C-NMR spectra of compound **3**

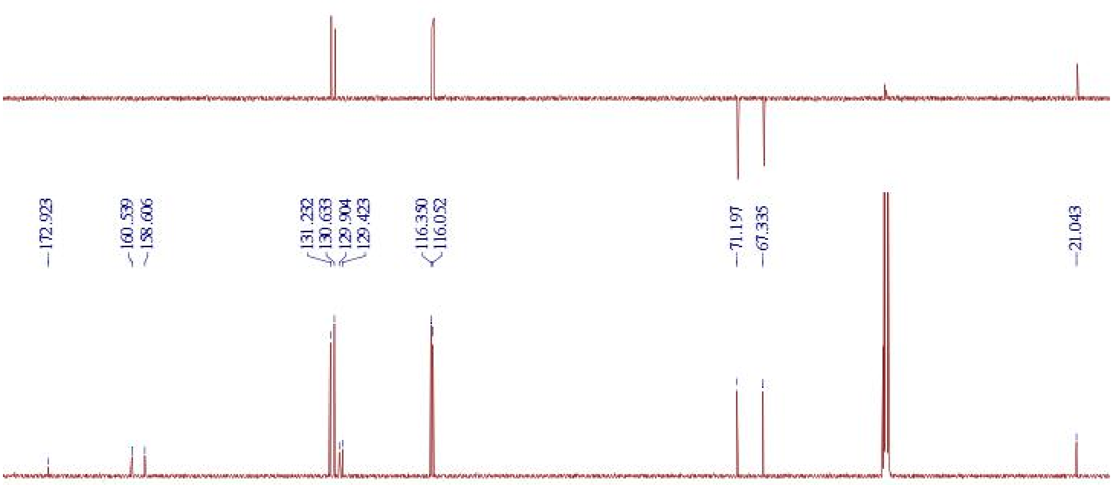

**S19.** HRESIMS spectra of compound **3**

| | | | | | |
|---|---|---|---|---|---|
| Error Margin (ppm): | 10 | DBE Range: | 0.0 - 20.0 | Electron Ions: | both |
| HC Ratio: | unlimited | Apply N Rule: | yes | Use MSn Info: | no |
| Max Isotopes: | all | Isotope RI (%): | 1.00 | Isotope Res: | 10000 |
| MSn Iso RI (%): | 75.00 | MSn Logic Mode: | AND | Max Results: | 500 |

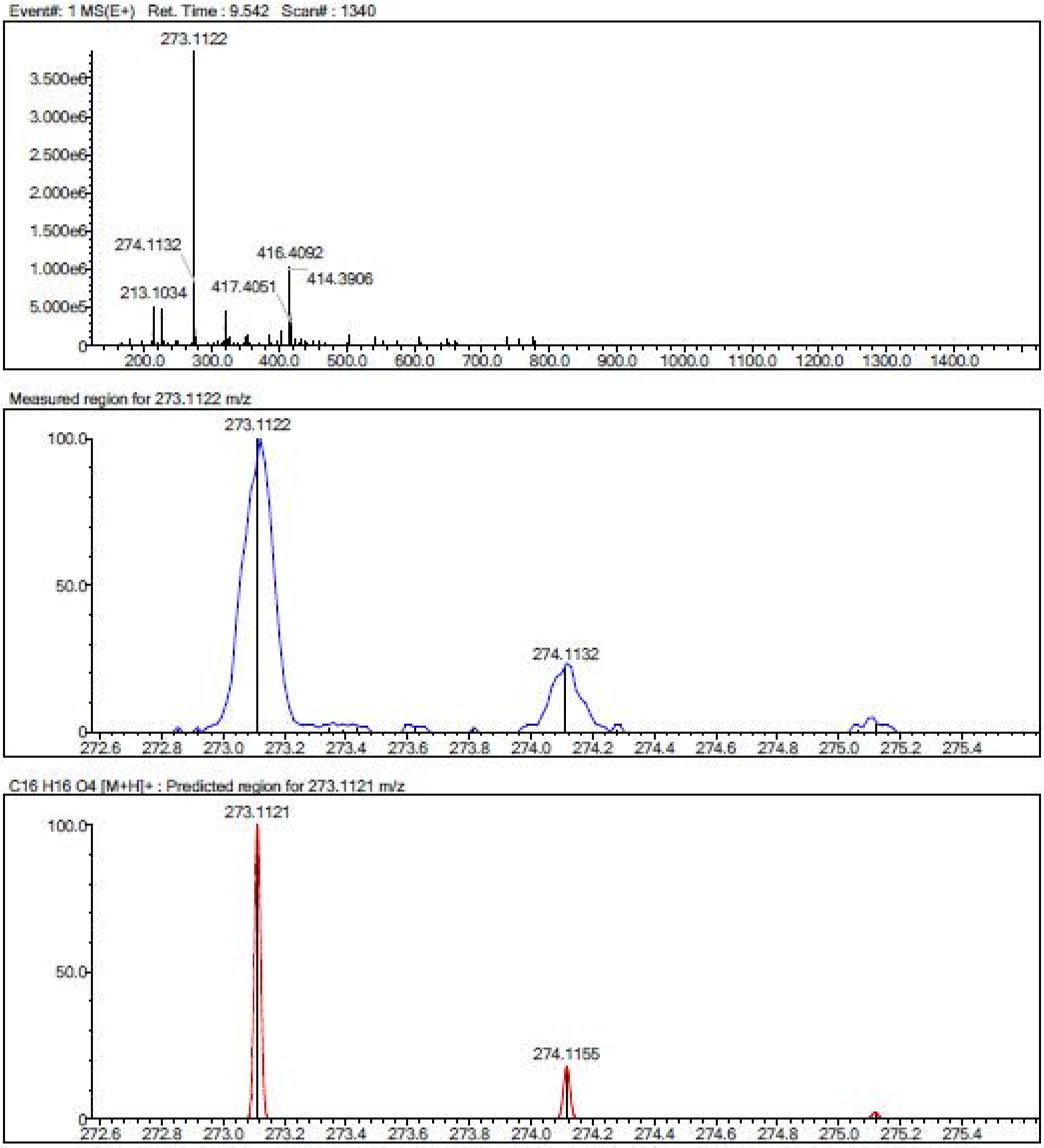


| Rank | Score | Formula (M) | Ion | Meas. m/z | Pred. m/z | Df. (mDa) | Df. (ppm) | Iso | DBE |
|---|---|---|---|---|---|---|---|---|---|
| 1 | 42.21 | C16 H16 O4 | [M+H]+ | 273.1122 | 273.1121 | 0.1 | 0.37 | 42.21 | 9.0 |

**S20.** UV spectra of compound **3**

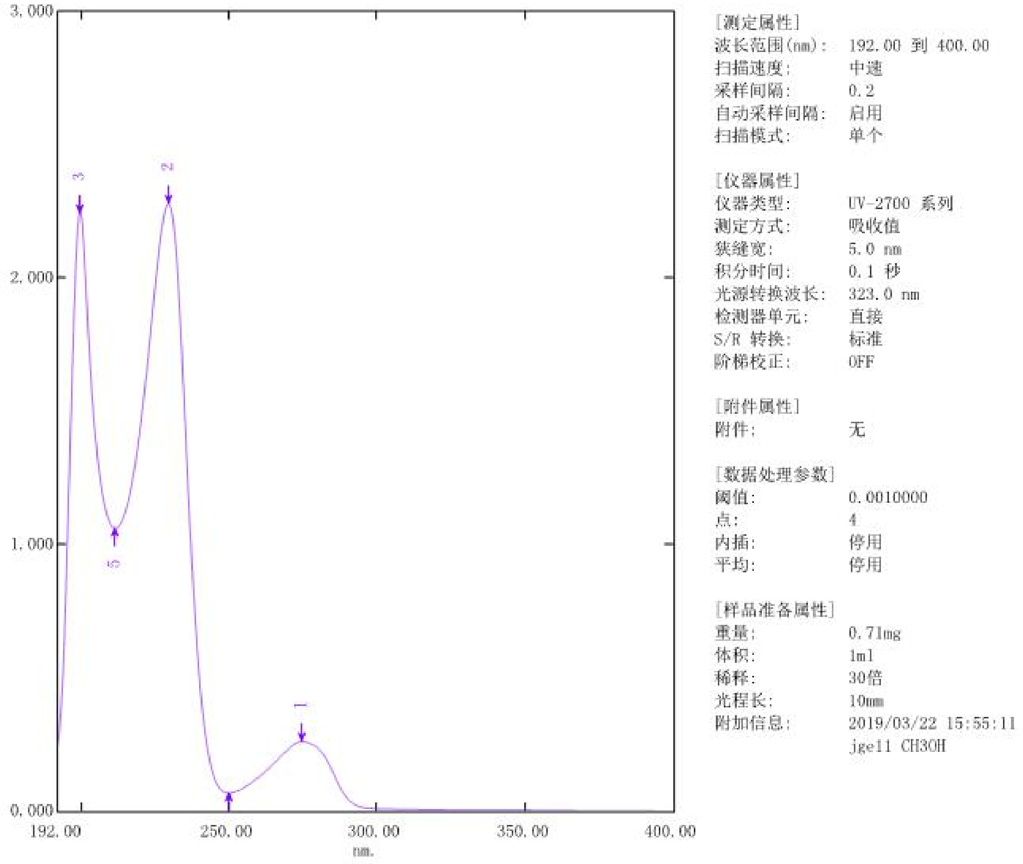


**S21.** IR spectra of compound **3**

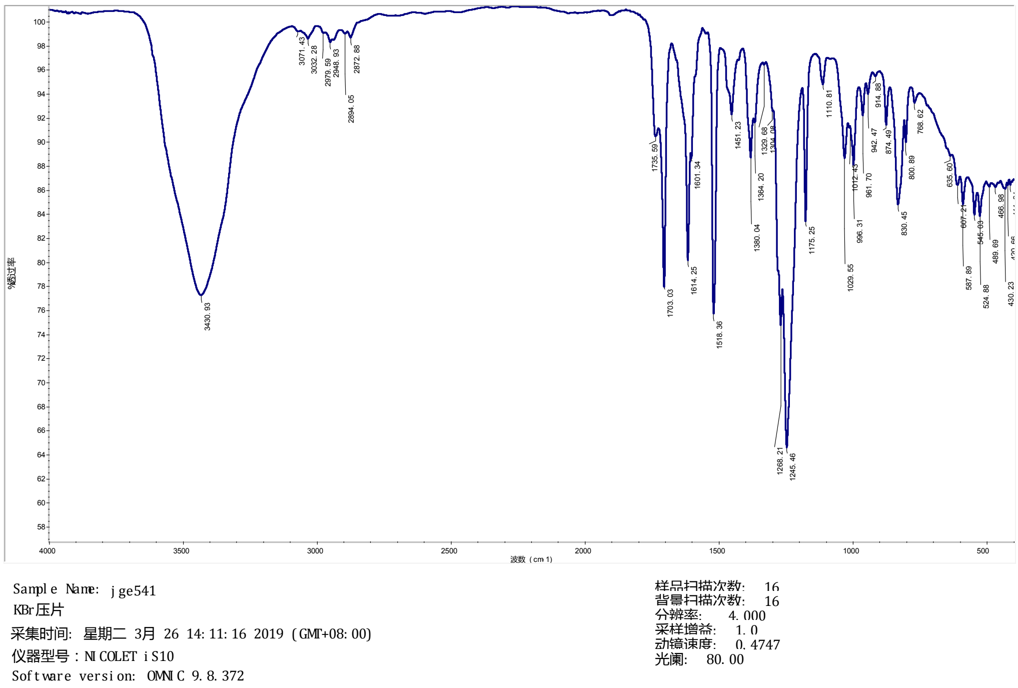

Supplement: Supplementary file 1 — Supplementary material 1 (PDF 835 kb) [file 13659_2019_213_MOESM1_ESM.pdf]
